# Utility of InTray COLOREX Screen agar and InTray COLOREX ESBL agar for urine culture in the Lao PDR

**DOI:** 10.1093/jacamr/dlac006

**Published:** 2022-02-07

**Authors:** Tamalee Roberts, Joy Silisouk, Davanh Sengdatka, Bountoy Sibounheuang, Ranoy Seljuk, Xao Vang, Amphonesavanh Sengduangphachanh, Viengmon Davong, Manivanh Vongsouvath, Nada Malou, Cecilia Ferreyra, Elizabeth A. Ashley, Andrew J. H. Simpson

**Affiliations:** 1 Lao-Oxford-Mahosot Hospital-Wellcome Trust Research Unit (LOMWRU), Microbiology Laboratory, Mahosot Hospital, Vientiane, Lao PDR; 2 Centre for Tropical Medicine and Global Health, Nuffield Department of Medicine, University of Oxford, Oxford, UK; 3 FIND, Geneva, Switzerland

## Abstract

**Background:**

There is a need for simple microbiology diagnostics to enable antimicrobial resistance surveillance in low- and middle-income countries.

**Objectives:**

To investigate the field utility of InTray COLOREX plates for urine culture and ESBL detection.

**Methods:**

Clinical urine samples from Mahosot Hospital, Vientiane, Lao PDR were inoculated onto chromogenic media and InTray COLOREX Screen plates between June and August 2020. Urine and isolates from other clinical specimens were inoculated onto COLOREX ESBL plates. A simulated field study investigating the field utility of the InTray COLOREX plates was also completed.

**Results:**

In total, 355 urine samples were inoculated onto standard chromogenic agar and InTray COLOREX Screen plates, and 154 urine samples and 54 isolates from other clinical specimens on the COLOREX ESBL plates. Growth was similar for the two methods (COLOREX Screen 41%, standard method 38%) with 20% discordant results, mainly due to differences in colony counts or colonial appearance. Contamination occurred in 13% of samples, with the COLOREX Screen plates showing increased contamination rates, potentially due to condensation. ESBL producers were confirmed from 80% of isolates from the COLOREX ESBL plates, and direct plating provided rapid detection of presumptive ESBL producers. *Burkholderia pseudomallei* also grew well on the ESBL plates, a relevant finding in this melioidosis-endemic area.

**Conclusions:**

The InTray COLOREX Screen and ESBL plates were simple to use and interpret, permitting rapid detection of uropathogens and ESBLs, and have the potential for easy transport and storage from field sites and use in laboratories with low capacity.

## Introduction

Urinary tract infection (UTI) is the most common extra-intestinal infection in women worldwide, and perhaps one of the most formidable challenges in clinical practice given its high prevalence, frequent recurrence and myriad of associated morbidities in the setting of rapidly evolving antimicrobial resistance.^1,2^ Most UTIs worldwide are treated on an empirical basis; thus empirical treatment should be based on available local data regarding the susceptibility of common pathogens to antibiotics. Such data are frequently lacking in low- and middle-income countries (LMICs), where antimicrobial resistance (AMR) surveillance systems are scarce and antibiotic self-medication is common with the potential for increased antibiotic resistance.^3^ High rates of resistance of *Escherichia coli* to commonly used antibiotics (amoxicillin, trimethoprim/sulfamethoxazole and quinolones) have been described.^4,5^ There has also been an increase in ESBLs seen in the Southeast Asia region with increases in ESBL-producing *E. coli* in blood cultures and urines seen over time.^6–8^ AMR is a critical public health issue both in high- and low-resource settings, but accurate surveillance for AMR requires clinical microbiology laboratories capable of culture and AST.^9^ The lack of laboratory capacity in many low-resource settings, which, if available, is often found in large cities only, has hindered our understanding of the prevalence and dynamics of antimicrobial resistance. This also limits both adequate patient care and knowledge of regional resistance patterns, which in turn can inform empirical therapy guidelines and aid antibiotic stewardship.^10^

Midstream urine culture from symptomatic patients for the detection of pathogens and AST remains the gold standard for the diagnosis of UTIs but is relatively slow (48–72 h) and requires access to a clinical laboratory. This results in delays to confirmation of adequate empirical antibiotic prescription. Though urine dipsticks are fast and amenable to point-of-care testing,^11^ a negative dipstick result is insufficient to rule out a UTI and they do not provide a confirmed microbiological diagnosis or AST results.^12^

The use of chromogenic media for processing urine specimens in many diagnostic laboratories has reduced the time to pathogen identification, however this labour-saving microbiological method, like several others, is not widely available in laboratories in LMICs because it is more expensive than traditional culture media.

Pre-prepared, commercially available culture media such as the InTray COLOREX Screen and ESBL agar plates (Biomed Diagnostics) have the potential to aid primary sample processing and transport to a referral laboratory, improve turnaround times for final result reporting to clinicians and aid early detection of ESBL-producing organisms.^13^ The InTray media are small, sealable commercially produced agar plates, similar to media used commonly in many microbiology laboratories worldwide, but designed for inoculation in clinics or remote sites prior to transport to testing laboratories. Like other chromogenic media, different bacterial species produce different coloured colonies, allowing rapid presumptive identification. A recent study in Zimbabwe on the utility of the InTray COLOREX Screen and ESBL plates showed growth was seen on the plates within 5–10 h of incubation, which allowed for rapid identification and a reduction in time of 60% to reporting of results.^13^

This study was designed to assess the effectiveness of the InTray COLOREX Screen plates for routine bacterial isolation from clinical urine samples compared with standard methods, plus the utility of InTray COLOREX ESBL plates for early ESBL detection. Operational stability of the COLOREX Screen plates in simulated field conditions was also assessed.

## Methods and materials

### Comparison of InTray COLOREX Screen plates with standard methods

The study included all sequential urine samples from inpatient and outpatients submitted routinely between June and August 2020 to the Microbiology Laboratory at Mahosot Hospital, Vientiane, Lao PDR (Laos), a tertiary government hospital with approximately 350 beds. Urine samples were inoculated (1 μL) onto chromogenic agar plates (*Brilliance* UTI Clarity agar, Oxoid) according to local standard operating procedures (SOP). In addition, all samples were inoculated (1 μL) onto InTray COLOREX Screen plates (Biomed Diagnostics) and were incubated aerobically at 35°C–37°C for 18–24 h before inspection for growth. All plates were re-incubated for a further 24 h if there was no growth. Chromogenic and COLOREX Screen plates were examined independently by different readers and colony counts (cfu/mL) and colonial appearance recorded on a worksheet and entered into Microsoft Excel. Different colony counts or organisms were recorded as discordant results. Organisms were presumptively identified according to the manufacturer’s colour chart (*E. coli* dark pink to reddish; *Enterococcus* sp. turquoise blue; *Klebsiella, Enterobacter* and *Citrobacter* metallic blue; *Proteus mirabilis* brown with halo; *Staphylococcus aureus* golden, opaque and small; *Staphylococcus saprophyticus* pink, opaque and small). Significant isolates were identified following standard microbiological procedures, including phenotypic testing. Antimicrobial susceptibility testing followed the EUCAST guidelines, version 10.0 (2020) with isolates recorded as susceptible, susceptible increased exposure, or resistant on worksheets and then entered into Microsoft Excel.

### InTray COLOREX ESBL plates

Suspected *E. coli* (pink/red colonies) or coliforms (dark blue/purple colonies) from the COLOREX Screen plates were sub-cultured onto COLOREX ESBL plates. In the latter part of the study, all urine samples were also directly plated onto the COLOREX ESBL plates as well as several isolates from other clinical sample types to test the feasibility of using the COLOREX ESBL plates to screen for ESBLs. Isolates were recorded as growth or no growth, and colony colour. ESBL confirmation testing was performed on isolates which grew, using the double-disc method (cefotaxime* *±* *clavulanate and ceftazidime* *±* *clavulanate [BD]). For the purpose of evaluation, all isolates that grew on the COLOREX ESBL plates, including oxidase-positive isolates, were worked up, in order to assess which organisms would grow on the media. Colonies growing on the media with the correct colour but with ESBL confirmation test negative were considered false positives.

### Simulated field study

To assess field utility and stability, a small number of urine samples were also inoculated onto COLOREX Screen plates as above and stored in a box at room temperature overnight, to simulate field conditions. These urines were also set up following the standard microbiological methods on chromogenic agar and incubated aerobically at 35°C–37°C for 18–24 h before inspection for growth. Growth was recorded the following morning. The COLOREX Screen plates were then incubated in air at 35°C–37°C overnight, re-examined the next morning and compared with standard study methods as above.

### Quality control

COLOREX Screen plates had quality control testing using *E. coli* ATCC 25922, *S. aureus* ATCC 25923 and a previously identified clinical *Enterococcus* sp. isolate, while the COLOREX ESBL plates were tested using *E. coli* ATCC 25922 and a previously confirmed ESBL-positive clinical isolate. The InTray COLOREX ESBL plates expired on 5 June 2020, the week the study started, and therefore all samples were set up on expired plates, however, as quality control passed, the plates were used. We were unable to do quality control on each of the plates used for clinical samples due to the small size of the plates. Mahosot Hospital Microbiology Department participates in the UK National External Quality Assurance Scheme programme. Results are reported following the MICRO Checklist.^14^

### Data analysis

The sensitivity and specificity of the InTray COLOREX plates compared with the standard method used in the laboratory was determined. Repeat samples were not analysed in this study. Data were analysed using Stata version 14 (StataCorp, College Station, TX, USA). Comparison of culture positive proportions were assessed using the χ^2^ test.

### Data sharing

De-identified data are available on request from the corresponding author. Images of plates are available at figshare (doi: 10.6084/m9.figshare.17871767).

### Ethics

Research Ethics Committee approval was received from the Oxford Tropical Research Ethics Committee (OxTREC Reference number 006-07) and the Ethics Committee of the University of Health Sciences, Lao PDR.

## Results

A total of 355 urine samples were set up for the primary comparison study. One hundred and ninety-nine urine samples or isolates were set up on the InTray COLOREX ESBL plates. In addition, for the simulated field study, 37 urine samples were set up on additional InTray COLOREX Screen plates.

### InTray COLOREX Screen plates comparison

Forty-one percent (146/355) of urine samples had growth on the COLOREX Screen plates compared with 38% (135/355) on standard chromogenic agar (*P *= 0.399). Single organisms were isolated from 23% (83/355) and 19% (68/355) of specimens respectively (*P *= 0.169). Results are summarized in Table 1. The differences were mainly due to contamination. Very similar recovery rates for the two methods were found for coliforms and *Enterococcus* species. Mixed growth occurred from approximately 20% of samples for both plate types.

**Table 1.** Urine growth comparison of InTray COLOREX Screen plate and chromogenic agar (Oxoid) and the most common organisms isolated

Growth characteristic	InTray COLOREX Screen plate, *n*/*N* (%)	Chromogenic agar (Oxoid), *n*/*N* (%)	Number different
Growth	146/355 (41)	135/355 (38)	11
No growth	209/355 (59)	220/355 (62)	12
1 organism	83/355 (23)	68/355 (19)	15
2 organisms	49/355 (14)	51/355 (14)	2
3 organisms	13/355 (4)	14/355 (4)	1
>3 organisms	1/355 (0.3)	2/355 (0.6)	1
*E. coli*	52/355 (15)	51/355 (14)	1
*Klebsiella*/*Enterobacter* sp.	21/355 (6)	21/355 (6)	0
*Enterococcus* sp.	46/355 (13)	43/355 (10)	3
*Staphylococcus* sp.	58/355 (16)	45/355 (13)	13

*n*, number of plates with growth characteristic; *N*, number of plates inoculated; %, percentage of plates with growth characteristic.

Overall, 20% (70/355) of samples had discordant results (Table 1). Most differences were minor and due to colony variance for the same organism on the chromogenic agar (which was not generally seen on the COLOREX Screen plates) or differences in colony counts between the plates. Contamination was suspected to be the reason for 13% (9/70) of these samples (5/9 from the COLOREX Screen plate and 4/9 from chromogenic agar). There were 3% (2/70) of plates where there was more growth on the COLOREX Screen plate, possibly due to condensation on the plate during overnight incubation. *E. coli* was the most common pathogen isolated followed by *Enterococcus* species. *Staphylococcus* spp. were considered contaminants in all but one case as per local SOPs; they were more common on the InTray COLOREX Screen plates compared with chromogenic agar. Colony colours for the different organisms were similar for both media types and followed the manufacturer’s descriptions. Other organisms that grew on the COLOREX Screen plates in smaller numbers included *Burkholderia pseudomallei*, *Acinetobacter* sp., *Candida* sp., *Pseudomonas aeruginosa* and *Salmonella* spp., all of which appeared as white colonies. Compared with the chromogenic agar, the COLOREX Screen plates had 94.6% sensitivity and 89.4% specificity (for identification of bacterial species).

### InTray COLOREX ESBL plates

There were 199 samples set up on the COLOREX ESBL plates: 154 directly from urine samples, 36 sub-cultured from isolates grown on the COLOREX Screen plate and 9 sub-cultured from growth on blood agar. Results are summarized in Table 2. There were 28% (55/199) of cultures with growth on the COLOREX ESBL plate and 80% (44/55) had an ESBL confirmation test performed, of which 82% (36/44) were confirmed as ESBL positive; 75% (27/36) *E. coli*, 22% (8/36) *K. pneumoniae* and 3% (1/36) *P. mirabilis*. Eleven isolates did not have confirmation testing done for various reasons, including isolates of *Enterobacter* or *Acinetobacter* species (which do not have ESBL confirmation testing as per local SOPs), plates being too mixed to achieve single colonies and growth of a yeast (one plate) or *B. pseudomallei* (one plate). Although not all isolates were confirmed, direct plating from urine samples provided presumptive ESBL results 24 h earlier than the routine methods (growth from 15% (23/154) of urine specimens).

**Table 2.** Culture growth results from InTray COLOREX ESBL plates inoculated directly with urine or from bacterial isolates

	Number of plates	Plates with growth, *n*/*N* (%)	Plates with ESBL testing, *n*/*N* (%)	ESBL confirmed, *n*/*N* (%)
Set up directly from urine	154	23/154 (15)	16/23 (70)	13/16 (81)
Set up from isolate	45	32/45 (71)	28/32 (88)	23/28 (82)

*n*, number of plates with growth; *N*, number of plates inoculated; %, percentage of plates with growth.

Of the eight isolates that were ESBL confirmation test negative, two appeared to have breakthrough colonies with the isolates susceptible to all antibiotics (both *E. coli*), one isolate was ceftriaxone susceptible but cefpodoxime and cefoxitin resistant (*E. coli*), another was ceftriaxone resistant but cefpodoxime susceptible and another was both ceftriaxone and cefpodoxime resistant (*E. coli*). One isolate was resistant to all antibiotics including ceftriaxone, cefpodoxime, cefoxitin and meropenem (*E. coli*). Antibiotic susceptibility results were available for 21 urine sample isolates that grew on the COLOREX ESBL plates (Table S1, available as Supplementary data at *JAC-AMR* Online). One plate appeared to be affected by condensation during incubation, which may have increased the growth on the COLOREX ESBL plate, and the last plate unfortunately did not have AST results recorded.

### Simulated field study

For the simulated field study there were 37 urine samples set up on both the COLOREX Screen plates and standard chromogenic agar. The growth results were compared and summarized in Table 3. Seven samples had growth on the COLOREX Screen plate after overnight incubation at room temperature (25°C–28°C). Four samples grew the same organism as grown on the chromogenic agar plate, one sample had an *Enterococcus* sp. on the COLOREX Screen plate but had a mixed growth (*Enterococcus* sp. and *Staphylococcus* sp.) on the chromogenic agar, and one sample grew an *Enterococcus* sp. on the COLOREX Screen plate but an *E. coli* on the chromogenic plate. One sample grew a white Gram-negative rod (not further identifiable) on the COLOREX Screen plate (a probable contaminant that could be due to the condensation that was seen on the plastic cover) while there was no growth on the chromogenic agar.

**Table 3.** Simulated field laboratory study results from urine inoculated onto InTray COLOREX Screen plates after overnight incubation in a transport box, growth after further overnight incubation at 37°C and growth on chromogenic agar after standard inoculation and incubation

Growth characteristic	COLOREX Screen plate after overnight incubation in box at room temperature, *n*/*N* (%)	Extra growth on COLOREX Screen plate after further overnight incubation at 37°C, *n*/*N* (%)	Chromogenic agar after overnight incubation (37°C), *n*/*N* (%)
Growth	7/37 (19)	13/37 (35)	14/37 (38)
No growth	30/37 (81)	17/37 (46)	23/37 (62)
1 organism	4	12	11
2 organisms	3	1	2
3 organisms	0	0	1
*E. coli*	4	3	8
*Klebsiella* sp.	1	0	0
*Enterococcus* sp.	2	4	5
*Staphylococcus* sp.	1	6	3
*Streptococcus* sp.	0	0	1
*Pseudomonas* sp.	0	1	1
White GNR (contaminant)	1	0	0

*n*, number of plates with growth characteristic; *N*, number of plates inoculated; %, percentage of plates with growth characteristic; GNR, Gram-negative rod.

Seven of the eight samples that grew on the chromogenic agar plates but did not grow on the COLOREX Screen plate after overnight incubation at room temperature grew after further overnight incubation at 37°C. The six remaining samples with growth on the COLOREX Screen plate after incubation at 37°C but no growth on the chromogenic agar plate included one *E. coli*, four *Staphylococcus* sp. and one mixed with *Staphylococcus* sp. and *Klebsiella* sp.

### Ease of use

During group discussion with laboratory staff, the InTray COLOREX Screen and ESBL plates proved to be simple to use and were well received by laboratory technical staff. However, due to the relatively small size of the agar plates, it was sometimes difficult to see single colonies. The plastic seal did not properly seal in a small number of samples. Photos of examples of growth characteristics on the InTray COLOREX Screen plates are available on figshare (doi: 10.6084/m9.figshare.17871767).

## Discussion

Overall, the InTray plates gave similar results to standard laboratory methods using chromogenic agar, although with a slightly higher contamination rate. Direct plating from urines provided presumptive ESBL results 24 h earlier than routine methods. For countries with a high ESBL burden, the direct plating of urines onto ESBL plates would be beneficial for patient treatment, with clinicians potentially able to change antibiotic prescriptions within 24 h.

There were occasions when colony variance was seen on the chromogenic agar but not on the InTray plates and vice versa. *Staphylococcus* spp. often had varying sizes on chromogenic agar, but the colonies were more consistent on the InTray plates. However, whilst there was colony variance in size, the colours were consistent between the two different plates and with the manufacturer’s package insert descriptions. *B. pseudomallei* grew well (as white colonies) on the COLOREX Screen plates, which would be helpful for melioidosis endemic countries such as Laos,^15^ though extra safety measures will need to be taken in these endemic areas and could raise problems if laboratories do not have biosafety cabinets, as staff should not open the plates on the bench. *B. pseudomallei*, *Acinetobacter* sp., *P. aeruginosa* and *Candida* sp. colony colours were not described in the manufacturer’s package insert and could be included, particularly as they are common isolates in the Southeast Asian region and isolation of *B. pseudomallei* is always clinically significant.^15–18^ Full identification was not carried out on non-clinically significant isolates that grew on the plates due to our MALDI-TOF being out of service.

The InTray COLOREX ESBL plates cost US$35 per box or US$1.75 per plate. In the microbiology department at Mahosot Hospital, where media are made in house, the cost for a chromogenic agar plate including the agar and plastic plate is US$1.48. This however does not include the cost for autoclaving, sterility indicators, quality control of the media and staff salaries. In smaller laboratories with less capacity, the COLOREX Screen plates may be more desirable, especially if the costs are similar.

While the field simulation study was limited, it does show the feasibility of using the plates at field sites and shipping them to a central laboratory for further work-up and identification. A consideration would be whether the plastic seal is strong enough and the effect that condensation and movement would have on isolation of single colonies and accurate colony counts.

There were some minor technical issues with the InTray plates. If the plates were not completely dry before use there could be excess growth on the plates, which can make it difficult to isolate single colonies and it could look like more growth compared with the chromogenic agar. Condensation was seen on the top of the clear plastic cover after incubation, which occasionally affected colony growth. It was difficult on occasion to get a complete seal on the InTray plates, which also leads to condensation and may also affect the culture if the plate becomes contaminated during transportation.

Although the COLOREX ESBL plates inhibited the growth of almost all susceptible isolates, there were several ESBL-negative organisms that grew on the ESBL plates. This may be due to the lack of ability to differentiate ESBL-mediated resistance from other similar resistance mechanisms, such as AmpC production; however, the same treatment change would apply if the organism was an AmpC producer so the result would still be clinically useful. This has also been shown in previous studies with other ESBL-selective media, with the growth of non-ESBL isolates including AmpC and other penicillinase producers.^19–21^*Acinetobacter* sp. and *B. pseudomallei* both grew well on the ESBL plates.

In LMICs where a lack of dedicated staff to make media and do the relevant quality control may exist, the use of pre-made agar such as the InTray COLOREX plates can be beneficial and time saving. The plates are also easy to use and require minimal training for staff, making them ideal for low-capacity settings.

There were several limitations to this study. Expired COLOREX ESBL plates were used, however quality control passed so the plates continued to be used without noticeable issues. Full identification and susceptibility testing was only carried out on clinically significant isolates. Therefore, it is not possible to know the full list of organisms that can grow on the plates. The condensation on the plates did make it difficult to isolate single colonies and organisms may have been missed for further work-up.

### Conclusions

The InTray COLOREX Screen and ESBL plates are simple to use and gave similar results to standard methods for urine culture using chromogenic media. Common urinary pathogens grew well, as did *B. pseudomallei*, a common and important pathogen in Southeast Asia. These plates could play a valuable role in isolation of significant urinary pathogens and rapid detection of ESBL-producing organisms, particularly in resource-limited settings. The ESBL plates can also assist in AMR surveillance activities with minimal work or necessary trained staff.

The findings also show the potential utility of inoculating the InTray plates at field sites and shipping them to a central laboratory for further work-up and identification, without loss of organism viability, although contamination is a potential issue. Further study of field utility would be worthwhile.

## Supplementary Material

dlac006_Supplementary_Data

## Transparency declarations

None to declare.

For the purpose of open access, the author has applied a CC BY public copyright licence to any author accepted manuscript version arising from this submission.

## Supplementary data

Table S1 is available as Supplementary data at *JAC-AMR* Online.
