# Supplementary material for: Utility of InTray COLOREX Screen agar and InTray COLOREX ESBL agar for urine culture in the Lao PDR
Source: JAC Antimicrob Resist. 2022 Feb 7;4(1):dlac006. doi: 10.1093/jacamr/dlac006 (PMC8826549; doi:10.1093/jacamr/dlac006)
Supplement: dlac006_Supplementary_Data [file dlac006_supplementary_data.docx]

**Supplementary data**

**Table S1**. Antibiotic susceptibility results for 21 isolates from urine samples which grew on the InTray COLOREX ESBL plates

| **ESBL plate ID** | **ESBL confirmation test** | **Organism** | **CPD** | **CRO** | **FOX** | **MEM** | **AMP** | **AMC** | **AK** | **SXT** | **CN** | **CIP** |
| --- | --- | --- | --- | --- | --- | --- | --- | --- | --- | --- | --- | --- |
| 1 | Positive | *E. coli* | R | R | - | S | R | S | S | R | S | S |
| 2 | Positive | *E. coli* | R | R | - | S | R | S | S | R | S | R |
| 3 | Positive | *E. coli* | R | R | - | S | R | R | S | R | R | R |
| 4 | Positive | *K. pneumoniae* | R | R | - | S | R | R | S | R | R | R |
| 5 | Positive | *E. coli* | R | R | - | S | R | S | S | R | S | R |
| 6 | Positive | *K. pneumoniae* | R | R | - | S | R | S | S | R | R | - |
| 7 | Positive | *E. coli* | R | R | R | S | R | R | R | R | R | R |
| 8 | Positive | *E. coli* | R | R | R | S | R | R | S | R | R | R |
| 9 | Positive | *K. pneumoniae* | R | R | S | S | R | S | S | R | R | R |
| 10 | Positive | *E. coli* | R | R | S | S | R | S | S | R | S | R |
| 11 | Positive | *E. coli* | R | R | S | S | R | S | S | R | R | S |
| 12 | Positive | *E. coli* | R | R | S | S | R | S | S | R | S | S |
| 13 | Negative | *E. coli* | R | R | R | S | R | R | S | R | R | - |
| 14 | Negative | *E. coli* | S | S | S | S | R | S | S | S | S | S |
| 15 | Negative | *E. coli* | R | S | R | S | R | R | S | R | R | S |
| 16 | Negative | *E. coli*^*^ | R | R | R | R | R | R | S | R | R | R |
| 17 | Negative | *E. coli* | S | R | S | S | R | R | S | R | S | S |
| 18 | Not done | *Acinetobacter* sp. | - | - | - | I | - | - | R | R | R | R |
| 19 | Not done | *Pantoea* sp. | R | R | R | S | R | R | S | S | R | R |
| 20 | Not done | *Enterobacter* sp. | R | R | R | S | R | R | S | S | R | R |
| 21 | Not done | *Burkholderia pseudomallei*^**^ | - | - | - | S | - | I | - | I | - | - |

CPD= cefpodoxime; CRO= ceftriaxone; FOX= cefoxitin; MEM= meropenem; AMP= ampicillin; AK= amikacin; SXT= trimpethroprim- sulphamethoxazole; CN= gentamicin; CIP= ciprofloxacin. S= susceptible; I= susceptible increased exposure; R= resistant (EUCAST version 10.0, 2020)

^*^Carbapenemase test positive

^**^Ceftazidime intermediate
